# Similar improvements in patient-reported outcomes for non-specific low back pain patients with and without lumbar spinal stenosis symptoms following a structured education and exercise therapy program

**DOI:** 10.1186/s12891-023-06950-5

**Published:** 2023-10-25

**Authors:** James J. Young, Alice Kongsted, Jan Hartvigsen, Carlo Ammendolia, Rikke Krüger Jensen

**Affiliations:** 1 Schroeder Arthritis Institute, Krembil Research Institute, University Health Network, Toronto, Canada; 2 Centre for Muscle and Joint Health, Department of Sports Science and Clinical Biomechanics, University of Southern Denmark, Odense, 5230 Denmark; 3 Chiropractic Knowledge Hub, Odense, 5230 Denmark; 4 Rebecca MacDonald Centre for Arthritis and Autoimmune Diseases, Mount Sinai Hospital, Toronto, Canada; 5 Institute for Health Policy, Management and Evaluation, University of Toronto, Toronto, Canada

**Keywords:** Low back pain, Lumbar spinal stenosis, Exercise therapy, Education

## Abstract

**Background:**

People with nonspecific low back pain (NSLBP) can also experience overlapping symptoms of lumbar spinal stenosis (LSS), but the impact on treatment outcomes is unknown. This study investigated differences in treatment outcomes for disability, back pain intensity, and leg pain intensity following an education and exercise therapy program for NSLBP patients with and without comorbid LSS symptoms.

**Methods:**

This was a longitudinal analysis of 655 Danish participants in the GLA:D® Back program; an education and exercise therapy program for people with persistent NSLBP. Participants were classified as having comorbid LSS symptoms based on self-report. Linear mixed models were used to assess differences in change in disability (Oswestry Disability Index [0-100]) and back and leg pain intensity (Numeric Rating Scale [0–10]) at 3-, 6-, and 12-months between those with and without LSS symptoms.

**Results:**

28% of participants reported LSS symptoms. No certain differences in change in disability or back pain intensity improvement were observed at any time-point between those with and without LSS symptoms. Participants with LSS symptoms had slightly greater improvement in leg pain intensity at 6- (-0.7, 95% CI -1.2 to -0.2) and 12-months (-0.6, 95% CI -1.2 to -0.1).

**Conclusion:**

Compared to those without LSS symptoms, patients with persistent NSLBP and LSS symptoms can expect similar improvements in disability and back pain intensity, and slightly greater improvements in leg pain intensity with treatment. Therefore, education and exercise therapy programs designed for NSLBP are likely helpful for those also experiencing LSS symptoms.

**Supplementary Information:**

The online version contains supplementary material available at 10.1186/s12891-023-06950-5.

## Background

Like nonspecific low back pain (NSLBP), lumbar spinal stenosis (LSS) is a common condition in the aging population [1], including in primary care settings [2]. Also like NSLBP, most patients with LSS can be managed at the primary care level with interventions including patient education and exercise therapy [1, 3]. At present a significant proportion of people seeking care for NSLBP have symptoms typically attributed to LSS [4], even if they have no formal LSS diagnosis, due to the well-documented overlap in symptoms and diagnostic uncertainty between NSLBP and LSS symptoms [5–10] and lack of commonly accepted diagnostic standards for LSS [1]. It is therefore of interest to know whether primary care patients with LSS symptoms benefit from programs designed for patients with NSLBP, or if they require interventions tailored specifically for LSS [11].

The GLA:D® Back program and patient registry presents the unique opportunity to evaluate the impact of LSS symptoms on outcomes following education and exercise therapy for people with NSLBP. GLA:D® Back is a standardised care package delivered across Denmark comprised of group-based patient education and exercise therapy aimed at improving self-management abilities [12, 13]. An objective of the GLA:D® Back research program is to identify subgroups of people who do not benefit sufficiently from the intervention [12] and previous work has found that 71% of participants in GLA:D® Back report “sometimes having pain or numbness in one or both legs or buttocks”; a symptom commonly attributed to LSS [4]. Other common LSS symptoms ranged in prevalence from 11 to 58% in these participants [4], but the impact on GLA:D® Back treatment outcomes has not been evaluated.

The objective of this study was to investigate differences in treatment outcomes for disability, back pain intensity, and leg pain intensity following an education and exercise therapy program (GLA:D® Back) for NSLBP patients with and without comorbid LSS symptoms. We hypothesised that LSS symptoms would be associated with less improvement in all outcomes.

## Methods

### Study design

Longitudinal analysis of registry data from the GLA:D® Back program for NSLBP [12]. GLA:D® Back consists of two patient education sessions and 8 weeks of supervised group exercise sessions [12, 14]. Detailed information on program content is available elsewhere [12–14]. This report conforms to the STROBE statement for reporting observational studies. As part of the larger GLA:D Back research program [12], ethical approval for this analysis was not required according to the Regional Committees on Health Research Ethics for Southern Denmark (S-2017000-93), but was conducted in accordance with the Declaration of Helsinki. All participants provided informed consent prior to enrolment in GLA:D® Back.

### Participants

People seeking care for persistent or recurrent NSLBP are eligible for GLA:D® Back if they are 18 years of age or older, understand Danish, and have a need for improved self-management based on shared decision-making by participant and enrolling clinician [12, 14]. Clinicians are given guidance in the GLA:D® Back training course on who may be best suited for the program, but are free to follow their best clinical judgement in a dialogue with the patient. Therefore, although the program was designed for NSLBP, clinicians can enrol patients with symptoms that overlap with symptoms of LSS and/or radiculopathy, when they deem the program relevant to these patients.

Self-report symptom items associated with LSS were included in the routinely collected electronic baseline survey in the GLA:D® Back registry. Participants with complete baseline LSS symptom data, who were enrolled between May and October 2019, were included in this analysis. This data collection period was selected to ensure only participants completing the treatment program (approximately 3 months) prior to the COVID-19 pandemic were included.

### LSS symptoms (exposure)

Participants were classified as having LSS symptoms (yes/no) if they reported all of the following: (1) “sometimes feeling pain or numbness in one/both legs or buttocks”; (2) one or more symptom-worsening activities (walking, standing for a while); (3) one or more symptom-relieving activities (bending forwards, sitting, riding a bicycle, bending over a shopping cart); and (4) were aged 60 years or older. While not diagnostic, these items are commonly-used in the self-report of LSS and are able to adequately differentiate leg pain from LSS from other sources of back-related leg pain [5]. Similar LSS definitions have been used in recent LSS studies [15–18].

### Outcomes

The primary outcome was the difference in mean change in disability between those with LSS symptoms and those without from baseline to 3-, 6-, and 12-months based on the Oswestry Disability Index (ODI) version 2.1 [19]. The ODI is scored from 0 (best) to 100 (worst) and is a valid and reliable measure in Danish people with NSLBP [19]. Although not specific to LSS, the ODI was selected as the primary outcome measure as it was relevant to both NSLBP and LSS.

Secondary outcomes were differences in mean change in back pain intensity and leg pain intensity, respectively, from baseline to 3-, 6-, and 12-months on the Numeric Rating Scale (NRS). The NRS is scored from 0 (best) to 10 (worst) for both back pain intensity and leg pain intensity [20].

### Analysis

#### Multiple imputation

Under the assumption of data missing at random, missing data for baseline covariates (confounders described in main analyses) and outcomes at all follow-up times were imputed using multiple imputation with chained equations [21]. The imputation model included all outcomes at each time point, LSS symptom status (exposure), and all confounders. Predictive mean matching was used to impute all continuous data, logistic regression for binary data, and ordered logistic regression for ordered categorical data. 52 imputed data sets were generated to match the proportion of missing data for the primary outcome at 12-months [21].

#### Main analyses

The primary outcome (difference in mean change in disability) from baseline to 3-, 6-, and 12-month follow-up) was estimated using a linear mixed model (restricted maximum likelihood ratio), where LSS symptom status and follow-up time were entered as fixed effects and participants nested within clinics were random effects. Unadjusted and adjusted differences in change with 95% confidence intervals (CI) were combined across imputed data sets using Rubin’s rules [22]. Potential confounders in the adjusted model included age (continuous), sex (binary), BMI (continuous), education level (ordered categorical), STarT Back Screening Tool classification (ordered categorical), and episode duration (ordered categorical). The same analysis approach was used for the secondary outcomes (difference in mean change in back pain intensity and leg pain intensity, respectively) from baseline to 3-, 6-, and 12-month follow-up. All analyses were performed in Stata 17.0.

#### Sensitivity analyses

In the first sensitivity analysis, the impact of missing data was explored by conducting a complete-case analysis, where only participants with completed outcomes were included. Adjusted differences (same controlled covariates as main analyses) in mean change in all outcomes from baseline to 3-, 6-, and 12-month follow-up were estimated.

Second, the impact of the selected age cut-point (≥ 60 years) in the LSS symptom definition was evaluated by repeating the analysis using an alternate cut-point of 50 years or older. This cut-point was selected since some LSS diagnostic studies suggest a cut-point as low as > 48 years [8] and 50 years old has been used in an analysis with similar LSS symptom items [6]. Adjusted differences (same controlled covariates as main analyses) in mean change in all outcomes from baseline to 3-, 6-, and 12-month follow-up were estimated.

## Results

### Sample characteristics

A total of 861 participants were enrolled in GLA:D® Back during the study period. A total of 206 participants were excluded due to missing baseline LSS symptom data. The excluded sample could not be compared with the analytic sample since the majority (99%) of excluded participants provided no baseline data. Accordingly, 655 participants were included in the analytic sample, of which 185 (28%) were classified as reporting LSS symptoms. Participants with LSS symptoms were substantially older, more often had a longer episode duration, and had worse disability, back pain, and leg pain scores when compared to those without LSS symptoms (Table 1).

**Table 1:** Overall participant baseline characteristics and by lumbar spinal stenosis symptom status

	All	LSS symptoms	**No LSS symptoms**
	(*n* = 655)	(*n* = 185)	(*n* = 470)
Age (years), mean (95% CI)	58.9 (57.9 to 60.0)	69.2 (68.3 to 70.1)	54.9 (53.8 to 56.1)
Missing, n	0	0	
Sex (female), % (95% CI)	71.8 (68.2 to 75.3)	70.7 (63.5 to 77.1)	72.3 (68.0 to 76.3)
Missing, n	9	1	8
BMI (kg/m^2^), mean (95% CI)	27.8 (27.4 to 28.2)	28.0 (27.2 to 28.8)	27.7 (27.2 to 28.2)
Missing, n	5	0	5
Education level, % (95% CI)			
No qualifying education	15.4 (12.7 to 18.4)	15.7 (10.8 to 21.7)	15.3 (12.2 to 18.9)
Vocational training	27.3 (23.9 to 30.9)	28.6 (22.3 to 35.7)	26.8 (22.9 to 31.1)
Higher education (2–4 years)	40.6 (36.8 to 44.5)	40.0 (32.9 to 47.4)	40.9 (36.4 to 45.4)
Higher education (> 4 years)	9.8 (7.6 to 12.3)	8.1 (4.6 to 13.0)	10.4 (7.8 to 13.5)
Other	4.7 (3.2 to 6.7)	4.9 (2.2 to 9.0)	4.7 (3.0 to 7.0)
Missing, n	14	5	9
STarT Back classification, % (95% CI)			
Low	43.8 (40.0 to 47.7)	34.1 (27.3 to 41.4)	47.7 (43.1 to 52.3)
Medium	25.3 (22.1 to 28.9)	31.4 (24.7 to 38.6)	23.0 (19.2 to 27.1)
High	28.1 (24.7 to 31.7)	31.9 (25.2 to 39.1)	26.6 (22.7 to 30.8)
Missing, n	18	5	13
Episode duration, % (95% CI)			
<4 weeks	7.6 (5.7 to 9.9)	4.9 (2.2 to 9.0)	8.7 (6.3 to 11.6)
4–12 weeks	11.5 (9.1 to 14.1)	7.6 (4.1 to 12.4)	13.0 (10.1 to 16.4)
3–12 months	20.8 (17.7 to 24.1)	18.9 (13.5 to 25.3)	21.5 (17.9 to 25.5)
>12 months	59.5 (55.7 to 63.3)	68.6 (61.4 to 75.3)	56.0 (51.3 to 60.5)
Missing, n	4	0	4
ODI, mean (95% CI)	26.0 (25.0 to 26.9)	30.2 (28.3 to 32.1)	24.3 (23.1 to 25.4)
Missing, n	12	3	9
Back pain, mean (95% CI)	5.6 (5.4 to 5.8)	6.0 (5.7 to 6.3)	5.5 (5.2 to 5.7)
Missing, n	2	0	2
Leg pain, mean (95% CI)	3.3 (3.1 to 3.6)	4.8 (4.4 to 5.1)	2.8 (2.5 to 3.0)
Missing, n	1	1	0

BMI = body mass index; ODI = Oswestry Disability Index (0 best to 100 worst); CI = confidence interval

### Missing outcome data

The proportion of missing ODI data was 32%, 42%, and 52% at 3-, 6-, and 12-month follow-up for the ODI. Proportion of missing back and leg pain NRS data was slightly less at each time-point. Participants with missing primary outcome data (ODI) at 12-months were younger, and had: a higher STarT Back classification (i.e., greater risk of chronicity), a longer episode duration length, and worse ODI and back and leg pain intensity scores (Additional File 1). A smaller proportion of participants with missing primary outcome data were classified as having LSS symptoms (24% versus 33%).

### Disability

**Fig. 1:**
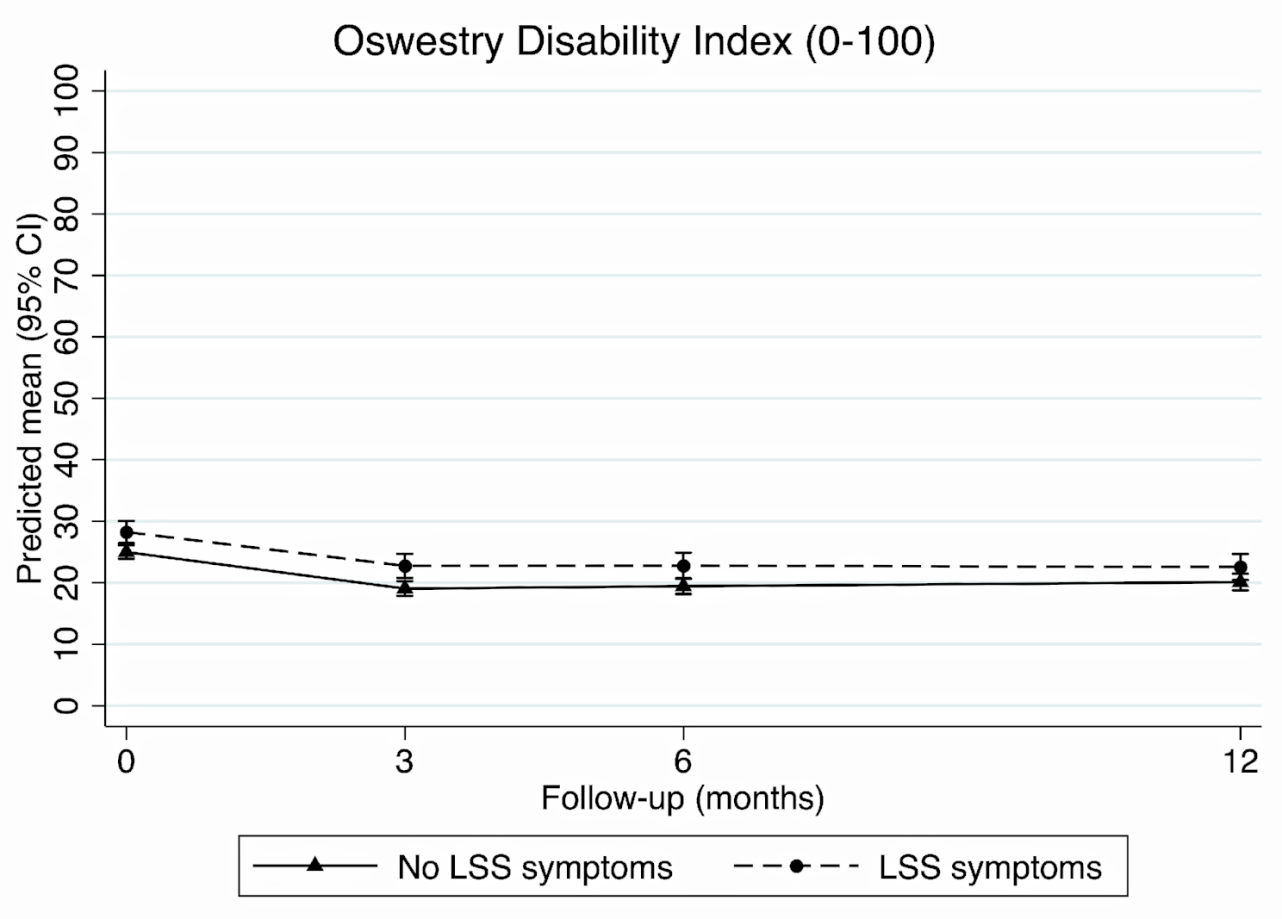
Change in disability scores at each time-point

In the adjusted analysis, we found disability scores slightly improved over time regardless of LSS symptom status, but those with LSS symptoms had slightly worse scores at baseline, 3-, and 6-months, but not at 12-months (Fig. 1). There was no certain difference between those with and without LSS symptoms in change in disability scores from baseline to 3- (0.4, 95% CI -1.4 to 2.3), 6- (0.1, 95% CI -2.0 to 2.2), or 12-month follow-up (-0.8, 95% CI -3.1 to 1.5) (Table 2). Unadjusted results were similar (Additional File 1).

**Table 2:** Adjusted difference in change in disability, back pain intensity, and leg pain intensity from baseline to 3-, 6-, and 12-month follow-up

	Baseline to 3-month follow-up		Baseline to 6-month follow-up		Baseline to 12-month follow-up	
	Mean change	Difference in mean change	Mean change	Difference in mean change	Mean change	Difference in mean change
ODI						
No LSS symptoms LSS symptoms	-6.0 (-6.9 to -5.0) -5.5 (-7.1 to -4.0)	--- 0.4 (-1.4 to 2.3)	-5.6 (-6.7 to -4.5) -5.5 (-7.2 to -3.7)	--- 0.1 (-2.0 to 2.2)	-4.9 (-6.0 to -3.7) -5.7 (-7.5 to -3.9)	--- -0.8 (-3.1 to 1.5)
Back NRS						
No LSS symptoms LSS symptoms	-1.8 (-2.1 to -1.6) -1.8 (-2.2 to -1.5)	--- -0.1 (-0.5 to 0.4)	-1.6 (-1.9 to -1.4) -1.8 (-2.3 to -1.4)	--- -0.2 (-0.7 to 0.3)	-1.6 (-1.9 to -1.3) -1.5 (-2.0 to -1.1)	--- 0.0 (-0.5 to 0.5)
Leg NRS						
No LSS symptoms LSS symptoms	-1.0 (-1.2 to -0.7) -1.3 (-1.7 to -0.9)	--- -0.3 (-0.8 to 0.2)	-0.9 (-1.1 to -0.6) -1.6 (-2.0 to -1.1)	--- -0.7 (-1.2 to -0.2)*	-0.7 (-1.0 to -0.4) -1.3 (-1.8 to -0.8)	--- -0.6 (-1.2 to -0.1)*

All results presented as mean and 95% confidence intervals. All models adjusted for age, sex, BMI, education level, STarT Back Screening Tool classification, and episode duration. ODI = Oswestry Disability Index (0 best to 100 worst); NRS = Numeric Rating Scale (0 no pain to 10 worst pain). * = between-group difference in change significant at 0.05 level

### Back pain intensity

**Fig. 2:**
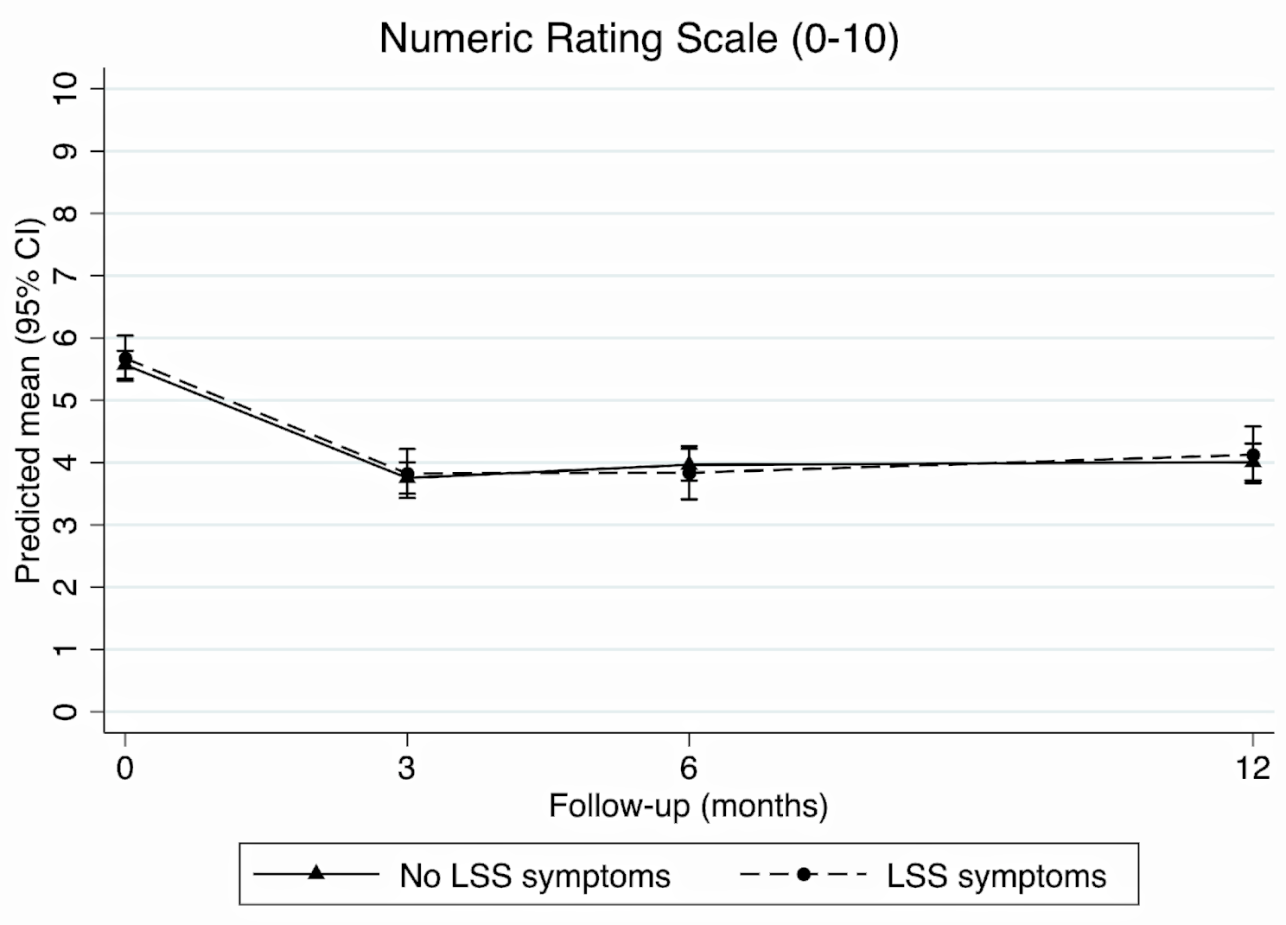
Change in back pain intensity scores at each time-point

The adjusted analysis found back pain intensity scores improved over time regardless of LSS symptom status and no certain differences in back pain intensity scores were found at any time-point (Fig. 2). There was no certain difference between those with and without LSS symptoms in change in back pain intensity scores from baseline to 3- (-0.1, 95% CI -0.5 to 0.4), 6- (-0.2, 95% CI -0.7 to 0.3), or 12-month follow-up (0.0, 95% CI -0.5 to 0.5) (Table 2). Unadjusted results were similar (Additional File 1).

### Leg pain intensity

**Fig. 3:**
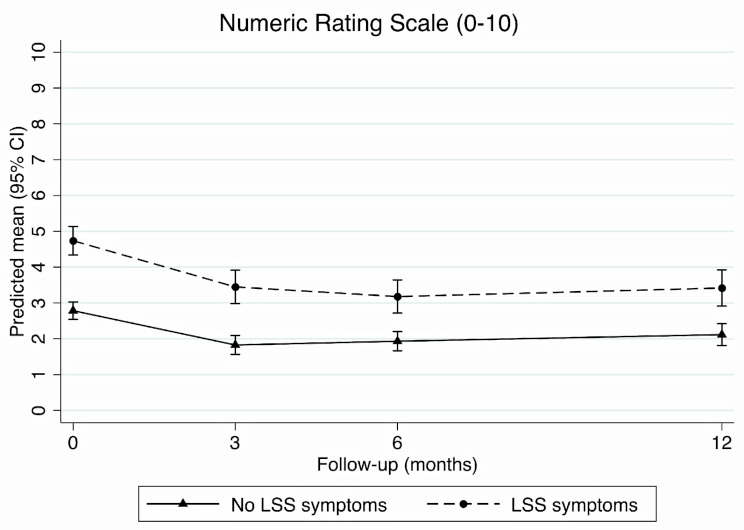
Change in leg pain intensity scores at each time-point

The adjusted analysis found leg pain scores intensity scores improved over time regardless of LSS symptom status, but those with LSS symptoms had worse leg pain intensity scores at all time-points (Fig. 3). There was no certain difference between those with and without LSS symptoms in change in leg pain intensity scores from baseline to 3-month follow-up (-0.3, 95% CI -0.8 to 0.2), but participants with LSS symptoms had slightly greater improvement from baseline to 6- (-0.7, 95% CI -1.2 to -0.2) and 12-month follow-up (-0.6, 95% CI -1.2 to -0.1) (Table 2). Unadjusted results were similar (Additional File 1).

### Sensitivity analyses

Results of the complete case sensitivity analysis (Additional File 1) and the alternate age cut-point sensitivity analysis (Additional File 1) confirmed that no certain difference in change in disability scores was observed between those with and without LSS symptoms at any time-point. Likewise, both sensitivity analyses confirmed the main analysis results for between-group differences in change in back and leg pain intensity scores, respectively.

## Discussion

Our findings suggest that regardless of LSS symptom status, patients with persistent or recurrent NSLBP can expect similar, small improvements in disability and moderate improvements in back pain intensity for up to a year following a structured education and exercise therapy program. However, patients with LSS symptoms can expect slightly greater improvement in leg pain intensity compared to those without LSS symptoms. Irrespective of differences in improvement, patients with LSS symptoms do experience slightly worse absolute disability and moderately worse absolute leg pain intensity scores both before and after treatment.

We found 28% of patients enrolled in primary care program for NSLBP also had LSS symptoms, which is in line with prevalence estimates in primary care settings [2]. These patients experienced similar magnitudes of improvements in all outcomes compared to patients without LSS symptoms, except for even greater leg pain intensity improvement from baseline to 6- and 12-months. However, it is unclear if the magnitude of these between-group differences (0.7 and 0.6 points on a 10-point scale, respectively) represent a clinically meaningful difference. While no studies have investigated what constitutes a meaningful between-group difference in leg pain intensity, previous studies suggest a minimum clinically important within-group change on the back pain NRS to range from 1.0- to 2.0-points in NSLBP populations [23, 24]. Since the 95% CI for both these between-group estimates include a 1.0-point change, there may be indication that a clinically meaningful difference in leg pain intensity improvement exists. The findings in both sensitivity analyses support this interpretation.

We are not aware of any previous studies that have investigated the impact of LSS symptoms in samples of patients participating in an education and exercise therapy program for persistent or recurrent NSLBP. Therefore, the findings of this study should be considered alongside relevant LSS literature [25] to guide treatment selection through a shared-decision making process by patients and clinicians. Our findings suggest that a program designed for patients with persistent or recurrent NSLBP can be helpful for patients with LSS symptoms, especially where primary care programs tailored for patients with LSS are not available. Treatment decision-making would benefit from trials making direct comparisons of programs like GLA:D® Back to LSS-specific programs that also include education and exercise, [16, 26] as well as other conservative treatment options for LSS [11].

The main study limitation is the LSS symptom case definition. There is no widely accepted diagnostic standard for LSS [1, 27] and varying definitions have been used in previous studies [8, 28]. We used symptom items identified in a review of self-report LSS screening items that found these items were able to differentiate leg pain due to LSS from other back-related causes of leg pain [5]. These items also include those most likely to identify LSS [8] and our definition resembles definitions used in a recent LSS trial and prevalence study [15, 16]. Confirmation of our results using the alternate age cut-point increases confidence in our findings. We were unable to include imaging confirmation of LSS since this is not collected in GLA:D® Back, but our approach is sufficient for preliminary estimates in this field [17, 18], considering LSS is a clinical diagnosis [27].

There was also a large proportion of missing baseline and outcome data, which is unavoidable in real-world implementation programs like GLA:D® Back. However, results were similar when analysing imputed outcomes and complete cases only, but the impact of differing baseline characteristics between the analytic and excluded samples are unknown. A selection bias in GLA:D® Back participants may also exist since enrolling clinicians are likely to recommend alternative treatments to patients with more severe clinical presentations of LSS, which may underestimate the impact of LSS symptoms on treatment outcomes. Conversely, between-group differences may be smaller than our observed results due to unmeasured confounding, for example from differences in comorbid pain sites and imaging characteristics such as LSS severity and number of affected levels.

## Conclusion

Compared to those without LSS symptoms, patients with persistent or recurrent NSLBP and LSS symptoms can expect similar improvements in disability and back pain intensity, and slightly greater improvements in leg pain intensity with treatment. Therefore, education and exercise therapy programs designed for people with NSLBP are likely helpful for those also experiencing LSS symptoms.

### Electronic supplementary material

Below is the link to the electronic supplementary material.

Additional File 1

## Data Availability

The datasets generated during and/or analysed during the current study are not publicly available due to licensing restrictions on availability of data but are available from the corresponding author on reasonable request.

## Abbreviations

NSLBP: Nonspecific low back pain
LSS: Lumbar spinal stenosis
STROBE: Strengthening the Reporting of Observational studies in Epidemiology
ODI: Oswestry Disability Index
NRS: Numeric Rating Scale
