# Supplementary material for: Similar improvements in patient-reported outcomes for non-specific low back pain patients with and without lumbar spinal stenosis symptoms following a structured education and exercise therapy program
Source: BMC Musculoskelet Disord. 2023 Oct 25;24:839. doi: 10.1186/s12891-023-06950-5 (PMC10599001; doi:10.1186/s12891-023-06950-5)
Supplement: Supplementary file 1 — Additional File 1 [file 12891_2023_6950_MOESM1_ESM.pdf]

Additional File 1

Supplementary Table 1. Baseline characteristics in participants with and without missing primary outcome data

	**Complete outcome data** (*n*=316)	**Missing outcome data** (*n*=339)
Age (years), mean (95% CI)	60.8 (59.5 to 62.1)	57.2 (55.7 to 58.7)
Missing, n	0	0
Sex (female), % (95% CI)	70.4 (65.0 to 75.4)	73.2 (68.1 to 77.9)
Missing, n	2	7
BMI (kg/m^2), mean (95% CI)	27.5 (26.9 to 28.1)	28.0 (27.4 to 28.6)
Missing, n	1	4
Education level, % (95% CI)		
No qualifying education	14.9 (11.1 to 19.3)	15.9 (12.2 to 20.3)
Vocational training	28.8 (23.9 to 34.1)	26.0 (21.4 to 31.0)
Higher education (2-4 years)	43.0 (37.5 to 48.7)	38.3 (33.1 to 43.8)
Higher education (>4 years)	8.9 (6.0 to 12.6)	10.6 (7.5 to 14.4)
Other	3.5 (1.8 to 6.1)	5.9 (3.6 to 9.0)
Missing, n	3	11
STarT Back classification, % (95% CI)		
Low	48.7 (43.1 to 54.4)	39.2 (34.0 to 44.7)
Medium	25.9 (21.2 to 31.2)	24.8 (20.3 to 29.7)
High	21.8 (17.4 to 26.8)	33.9 (28.9 to 39.2)
Missing, n	11	7
Episode duration, % (95% CI)		
<4 weeks	9.8 (6.8 to 13.6)	5.6 (3.4 to 8.6)
4-12 weeks	10.4 (7.3 to 14.4)	12.4 (9.1 to 16.4)
3-12 months	21.5 (17.1 to 26.5)	20.1 (15.9 to 24.7)
>12 months	57.3 (51.6 to 62.8)	61.7 (56.2 to 66.9)
Missing, n	3	1
ODI, mean (95% CI)	24.4 (23.0 to 25.8)	27.5 (26.1 to 28.8)
Missing, n	1	11
Back pain, mean (95% CI)	5.4 (5.1 to 5.6)	5.8 (5.6 to 6.1)
Missing, n	2	0
Leg pain, mean (95% CI)	3.1 (2.8 to 3.4)	3.5 (3.2 to 3.8)
Missing, n	0	1
LSS symptoms, % (95% CI)	33.2 (28.1 to 38.7)	23.6 (19.2 to 28.5)
Missing, n	0	0
LSS symptoms (alternate definition), % (95% CI)	43.0 (37.5 to 48.7)	36.0 (30.9 to 41.3)
Missing, n	0	0

Supplementary Table 2. Main analyses unadjusted results

	Baseline to 3-month follow-up		Baseline to 6-month follow-up		Baseline to 12-month follow-up	
	**Mean change**	**Difference in mean change**	**Mean change**	**Difference in mean change**	**Mean change**	**Difference in mean change**
**ODI**						
No LSS symptoms	-6.0 (-6.9 to -5.0)	---	-5.6 (-6.7 to -4.5)	---	-4.9 (-6.0 to -3.7)	---
LSS symptoms	-5.5 (-7.1 to -4.0)	0.4 (-1.4 to 2.3)	-5.5 (-7.2 to -3.7)	0.1 (-2.0 to 2.2)	-5.7 (-7.5 to -3.9)	-0.8 (-3.1 to 1.5)
**Back NRS**						
No LSS symptoms	-1.8 (-2.1 to -1.6)	---	-1.6 (-1.9 to -1.4)	---	-1.6 (-1.9 to -1.3)	---
LSS symptoms	-1.8 (-2.2 to -1.5)	0.0 (-0.5 to 0.4)	-1.8 (-2.3 to -1.4)	-0.3 (-0.7 to 0.3)	-1.5 (-2.0 to -1.1)	0.0 (-0.5 to 0.5)
**Leg NRS**						
No LSS symptoms	-1.0 (-1.2 to -0.7)	---	-0.9 (-1.1 to -0.6)	---	-0.7 (-1.0 to -0.4)	---
LSS symptoms	-1.3 (-1.7 to -0.9)	-0.3 (-0.8 to 0.2)	-1.6 (-2.0 to -1.1)	-0.7 (-1.2 to -0.2)*	-1.3 (-1.8 to -0.8)	-0.6 (-1.2 to -0.1)*

All results presented as mean and 95% confidence intervals. * = between-group difference in change significant at 0.05 level.

Supplementary Table 3. Complete case analyses results

	Baseline to 3-month follow-up		Baseline to 6-month follow-up		Baseline to 12-month follow-up	
	Mean change	Difference in mean change	Mean change	Difference in mean change	Mean change	Difference in mean change
**ODI**						
No LSS symptoms	-5.9 (-6.9 to -4.8)	---	-5.4 (-6.5 to -4.3)	---	-4.8 (-6.0 to -3.6)	---
LSS symptoms	-5.4 (-7.0 to -3.9)	-0.4 (-1.4 to 2.3)	-5.5 (-7.1 to -3.9)	-0.1 (-2.1 to 1.8)	-6.2 (-7.8 to -4.5)	-1.4 (-3.4 to 0.6)
**Back NRS**						
No LSS symptoms	-1.8 (-2.1 to -1.6)	---	-1.6 (-1.9 to -1.3)	---	-1.6 (-1.9 to -1.3)	---
LSS symptoms	-1.8 (-2.2 to -1.5)	0.0 (-0.5 to 0.4)	-1.9 (-2.3 to -1.5)	-0.3 (-0.8 to 0.2)	-1.5 (-2.0 to -1.1)	0.0 (-0.5 to 0.5)
**Leg NRS**						
No LSS symptoms	-1.0 (-1.3 to -0.7)	---	-0.8 (-1.1 to -0.5)	---	-0.7 (-1.0 to -0.4)	---
LSS symptoms	-1.2 (-1.6 to -0.8)	-0.2 (-0.7 to 0.3)	-1.6 (-2.0 to -1.2)	-0.8 (-1.3 to -0.2)*	-1.3 (-1.8 to -0.9)	-0.7 (-1.2 to -0.1)*

All results presented as mean and 95% confidence intervals. All models adjusted for age, sex, BMI, education level, STarT Back Screening Tool classification, and episode duration. * = between-group difference in change significant at 0.05 level.

Supplementary Table 4. Alternate age cut-point LSS symptom definition analyses results

	Baseline to 3-month follow-up		Baseline to 6-month follow-up		Baseline to 12-month follow-up	
	**Mean change**	**Difference in mean change**	**Mean change**	**Difference in mean change**	**Mean change**	**Difference in mean change**
**ODI**						
No LSS symptoms	-6.3 (-7.3 to -5.2)	---	-5.4 (-6.6 to -4.3)	---	-4.4 (-5.7 to -3.2)	---
LSS symptoms	-5.2 (-6.5 to -3.9)	1.1 (-0.6 to 2.8)	-5.7 (-7.2 to -4.2)	-0.3 (-2.2 to 1.6)	-6.1 (-7.7 to -4.6)	-1.7 (-3.8 to 0.4)
**Back NRS**						
No LSS symptoms	-1.8 (-2.1 to -1.6)	---	-1.6 (-1.9 to -1.3)	---	-1.6 (-1.9 to -1.3)	---
LSS symptoms	-1.8 (-2.1 to -1.5)	0.0 (-0.4 to 0.4)	-1.8 (-2.1 to -1.4)	-0.2 (-0.6 to 0.3)	-1.5 (-1.9 to -1.1)	0.1 (-0.4 to 0.6)
**Leg NRS**						
No LSS symptoms	-0.9 (-1.2 to -0.6)	---	-0.6 (-0.9 to -0.3)	---	-0.5 (-0.8 to -0.2)	---
LSS symptoms	-1.3 (-1.7 to -0.9)	-0.4 (-0.9 to 0.0)	-1.7 (-2.1 to -1.3)	-1.1 (-1.6 to -0.6)*	-1.4 (-1.8 to 1.0)	-0.8 (-1.4 to -0.3)*

All results presented as mean and 95% confidence intervals. All models adjusted for age, sex, BMI, education level, STarT Back Screening Tool classification, and episode duration. * = between-group difference in change significant at 0.05 level.
